# Evaluation of etoxazole against insects and acari in vegetables in China

**DOI:** 10.1093/jis/14.1.104

**Published:** 2014-08-01

**Authors:** Yongqiang Li, Na Yang, Xingcun Wei, Yun Ling, Xinling Yang, Qingmin Wang

**Affiliations:** 1 Key Laboratory of Pesticide Chemistry and Application, MOA, Department of Applied Chemistry, College of Science, China Agricultural University, People's Republic of China; 2 State Key Laboratory of Elemento-Organic Chemistry, Institute of Elemento-Organic Chemistry, Nankai University, People's Republic of China

**Keywords:** bioactivity, insect growth regulator, susceptible strain, resistant strain, acaricide/insecticide alternative

## Abstract

Etoxazole, 2-(2,6-difluorophenyl-4-[4-(1,1-dimethylethyl)-2-ethoxy-phenyl]-4,5-dihydrooxazole, an organofluorine chitin synthesis inhibitor, was assayed for its bioactivities against several major insect and acarus pests and compared to several other pesticides: two chitin synthesis inhibitors, hexaflumuron and chlorfluazuron; a pyrethroid, permethrin; an organophosphate, acephate; a carboximide, hexythiazox; and a tetrazine, clofentezine. The LC _50_ of etoxazole was calculated using probit analysis of the concentration-dependent mortality data against susceptible and resistant strains of the beet armyworm, *Spodoptera exigua* (Hubner) (Lepidoptera: Noctuidae); diamondback moth, *Plutella xylostella* L. (Plutellidae); bean aphid, *Aphis craccivora* Koch (Hemiptera: Aphididae); and carmine spider mite, *Tetranychus cinnabarinus* (Boisduval) Boudreaux (Trombidiformes: Tetranychidae). The resistant strains were found to be resistant against all tested pesticides except etoxazole. The bioactivity of etoxazole was many times that of the other tested insecticides and acaricides widely used in vegetable crops in China. On the basis of our research, etoxazole can be expected to be extensively used on vegetable crops in China.

## Introduction

Vegetables are a leading economic crop, and insects and acari have important impacts on yield and quality. In recent years, many new physical and biological control methods have been developed that favor the environment and beneficial organisms, such as biological control organisms, organic insecticides, and physical and horticultural activities. At present, due to restrictions imposed on agricultural and economic development, developing and applying new chemical insecticides and acaricides are still major measures for coping with insect and acari damage in vegetable production systems in China. However, the toxicity of chemical insecticides and acaricides is a primary drawback, as they are a hazard to the environment, human health, and beneficial organisms. Insects and acari have developed resistance to many pesticides because pesticides with a similar mechanism on targets were used repeatedly. Insect growth regulators are designed and synthesized to take advantage of unique aspects of development compared to other organisms ( Verloop et al. 1977 ; Oberlander et al. 1998 ), which makes them safe to nontargets, highly friendly to the environment, and selective for insects and acari. Therefore, IGRs have been developed in recent years by researchers throughout the world ( Qian 1996 ; Yang et al. 1999 ).

**Figure 1.**
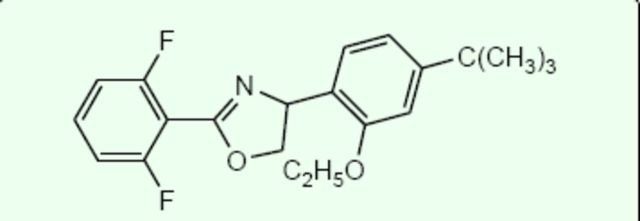
Chemical structure of etoxazole. High quality figures are available online.

Etoxazole ( Figure 1 ) was produced by Sumitomo Chemical in 1998 and developed as a new-generation insecticide and acaricide (Hirose et al. 1996; Yagi et al. 2000 ; Suzuki et al. 2001 , 2002; Tisdell et al. 2004 ). Etoxazole (trade name: Baroque Flowable; molecular mass of 359.42) is a white, free-flowing, crystalline powder that can be dissolved very easily in general organosolvents such as ethyl acetate, dimethylbenzene, tetrahydrofuran, cyclohexanone, acetone, and alcohol. Evaluation of its toxicity and behavior in the environment was done for the Standing Committee on the Food Chain and Animal Health belonging to the European Commission Health & Consumer Protection Directorate-General in 2004 ( Kyprianou 2005 ). Etoxazole is known as a biofriendly pesticide alternative to carbamates, organo-chlorines, and other acaricides and insecticides, the uses of which are strictly limited and even prohibited in some cases.

The mechanism of action of etoxazole (inhibition of the moulting process during insect and mite development) is similar to that of benzoylphenylureas ( Lee et al. 2004 ; Nauen et al. 2006 ; Asahara et al. 2008 ; Sun et al. 2008 ), a class of insecticides known to interfere with chitin biosynthesis. However, benzoylphenylureas as insect growth regulators have some shortcomings, such as a quite narrow spectrum of efficacy only on Lepidoptera and a high acute toxicity to nontarget insects ( Sun et al. 2009 ).

The rotational application of compounds with different modes of action in order to prevent or delay the rapid development of resistance is one of the major techniques used in resistance management strategies. The nervous system is the most commonly targeted site of the current main pesticides against insects and acari, and so far, little cross-resistance between them and etoxazole has been reported. Although it has been almost ten years since etoxazole was publicly introduced in 1994 and launched in 1998 as an acaricide/insecticide, its insecticidal and acaricidal activities have not yet been evaluated in detail ( Ishida et al. 1994 ). Moreover, its bioactivity against pesticide-resistant insects and acari collected in the field had not been reported in China.

The beet armyworm, *Spodoptera exigua* (Hübner) (Lepidoptera: Noctuidae); diamondback moth, *Plutella xylostella* L. (Plutellidae); bean aphid, *Aphis craccivora* Koch (Hemiptera: Aphididae); and the carmine spider mite, *Tetranychus cinnabarinus* (Boisduval) Boudreaux (Trombidiformes: Tetranychidae) were used in this research because they are several of the most important pests of vegetables and are widely distributed in China. Laboratory colonies of these organisms were raised without pesticide use. Field-collected insecticide-resistant colonies were also established. In this paper, we report on the bioactivity of etoxazole against these insects and spider mites and compare the results with those of several pesticides. The purpose of our research is to ascertain the bioactivity of etoxazole as an alternative pesticide against pests in vegetable crops and to evaluate its application prospects.

## Materials and Methods

### Chemicals

Etoxazole (purity 99%) was synthesized by our group using the method of Luo and Yang (2007) . 5% Etoxazole emulsifiable concentrate (EC) was prepared by our group. Hexaflumuron (purity 99%) was purchased from Shandong Yucheng Pesticide Biochemical Co., Ltd. Chlorfluazuron (purity 98%) was purchased from Shijiazhuang Jitai Sanmu Pesticide Chemical Industry Co., Ltd. Permethrin (purity 95%) was purchased from Jiangsu Su-hua Group Co., Ltd. Acephate (purity 95%) was purchased from Lianyungang Dongjin Chemical Co., Ltd. Hexythiazox (purity 98%) was purchased from Shanghai Dibai Plant Protection Co., Ltd. Clofentezine (purity 98%) was obtained from Shijiazhuang Lufeng chemical Co., Ltd. 5% Chlorfluazuron EC was purchased from Ishihara Sangyo Kaisha Ltd. 30% Acephate EC was purchased from Lianyungang Dongjin Chemical Co., Ltd. 5% Hexythiazox EC was purchased from Shanxi Kexing Pesticide Liquid Fertilizer Co., Ltd. Tween 20 and dimethyl sulfoxide (DMSO) were purchased from Alfa Aesar China (Tianjin) Co., Ltd.

### Insect strains

All insecticide-susceptible strains used in the study were reared in the bioassay platform of the State Key Laboratory of Elemento-Organic Chemistry, Nankai University, China. A susceptible strain of *S. exigua* was obtained from the College of Life Science, Nankai University, and reared in isolation under standard laboratory conditions of 27 ± 1ºC, 50∽75% RH, 14:10 L:D, and no exposure to any insecticides for several years. A susceptible strain of *P. xylostella* was reared on cabbage plants under standard laboratory conditions without exposure to chemicals for the past ten years. A susceptible strain of *A. craccivora* has been kept under laboratory conditions of 20 ± 1ºC, 40%∽60% RH, natural illumination, without any exposure to insecticides, since the model of screening for new compound’s bioactivity to bean aphids was established in 1990s ( Wakwmura 1988 ; Goh et al. 1991 ; Raymond et al. 1994 ). Resistant strains of *S. exigua, P. xylostella,* and *A. craccivora* were collected in the Zhangjiawo vegetable production area, Xiqing district, Tianjin city, China.

### Acarus strains

A susceptible strain of *T. cinnabarinus* was reared in the conservatory of the bioassay platform of State Key Laboratory of Elemento-Organic Chemistry, Nankai University, with the standard conditions of 24 ± 2ºC, dry air and good aeration, with natural illumination and without exposure to any acaricides. To obtain mite eggs, 20 adult spider mites were placed on a leaf of the common bean plant, *Phaseolus vulgaris* L. (Fabales: Fabaceae), for 24 hours. The adult mites were removed after 80 eggs had been laid. To obtain young mites, the mite eggs were allowed to develop on the leaves for 6 days. Then, the leaves with young mites were placed on leaves of the test plants. The cultured conditions of adult mites, mite eggs, and young mites were the same ( Wang et al. 2010 ). A resistant strain of *T. cinnabarinus* was collected in the field of the Institute of Plant Protection, Tianjin Academy of Agricultural Science, Wuqing district, Tianjin city, China.

### Bioassay against *S. exigua* and *P. xylostella*

The bioactivity bioassay of etoxazole, hexaflumuron, and chlorfluazuron (including EC preparations) against *S. exigua* and *P. xylostella* were tested by the leaf-dip method. For each test sample, a stock solution at a concentration of 200 mg·L ^-1^ in DMSO was prepared and then diluted to the required series concentrations with water containing Tween-20. Leaf disks (5 cm × 1 cm) from fresh cabbage leaves were dipped into the test solution for 10 sec. After air-drying on a filter paper, the leaf disks were treated with the test compound and then placed individually into Petri dishes (7 cm diameter). Second-instar larvae were transferred individually into the Petri dishes. Infested leaves treated with water and DMSO were provided as controls. Six replicates (10 larvae per replicate) were performed. Percentage mortalities were evaluated four days after treatment in the culture conditions and corrected with Abbott’s formula ( Chen et al. 2007 ; Luo et al. 2007 ; Shang et al. 2010 ; Zhao et al. 2010 ).

### Bioassay against *A. craccivora*

The insecticidal activities of etoxazole, permethrin, and acephate (including EC) against *A. craccivora* were assayed by a slightly modified FAO dip test. A stock solution in DMSO of a concentration of 200 mg·L ^-1^ was prepared and then diluted to the required series concentrations with water containing Tween-20. Tender bean shoots with 60 healthy uniform apterous adults were dipped in the concentration series of the compounds for 5 sec and then air-dried. Infested leaves treated with water and DMSO were provided as controls. Each test was carried out in triplicate. The processes of all tests were performed under standard laboratory conditions. Percentage mortalities were evaluated four days after treatment and corrected with Abbott’s formula ( FAO 1979 ; Nauen et al. 2006 ).

### Bioassay against *T. cinnabarinus* mite eggs and young mites

The activity of etoxazole, hexythiazox, and clofentezine (including EC) against mite eggs and young mites was evaluated by the same procedure with a slightly modified FAO dip test. A stock solution in DMSO of a concentration of 200 mg·L ^-1^ was prepared and then diluted to the required series concentrations with water containing Tween-20. Spider mite eggs and young spider mites on leaves were prepared as described in acarus strains. There were about 80 spider mite eggs or young spider mites per leaf. The mite-infested plants were soaked in the series of compounds for 3 sec; then, the superfluous liquid was removed by shaking the plants. Infested leaves soaked in water and DMSO were provided as controls. Each test was carried out in triplicate. The processes of all tests were performed under standard laboratory conditions. After 10 days, the unhatched egg rates (%) were calculated and percentage mortality of young spider mites was evaluated and corrected using Abbott’s formula ( Kuhn et al. 1992 , 1993; Dai et al. 2008 ).

### Date analysis

After all percentage mortalities were corrected with Abbott’s formula, the results were expressed as the mean value of parallel experiments (Abbot 1925). That is to say, if the percentage mortality of the control was less than 5%, the result was directly used; but if the percentage mortality was less than 20%, the result was corrected by V=((X-Y)/X)*100 (V = value of corrected mortality, X = livability of the control, Y = livability of the treat), or the test was invalid. The LC _50_ value (median lethal concentration) was calculated using probit analysis of the concentration-dependent mortality dates performed with the statistical software DPS v. 7.05 ( Siegel 1988 ).

## Results and Discussion

### Effects of insecticides against *S. exigua*

**Figure 2.**
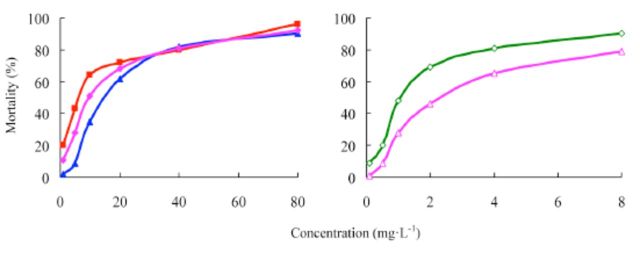
Concentration-response curves of etoxazole and other insecticides against *Spodoptera exigua* (susceptible). 99% etoxazole (A), 5% etoxazole EC ( ), 97% hexaflumuron ( ), 98% chlorfluazuron ( **▲** ), and 5% chlorfluazuron EC (♦). High quality figures are available online.

**Figure 3:**
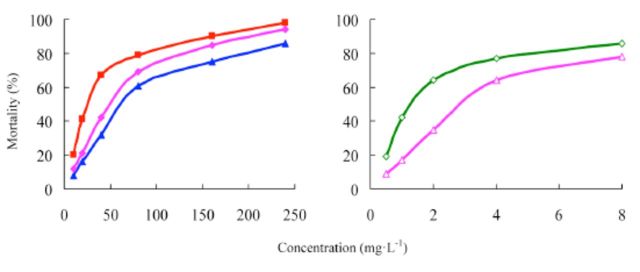
Concentration-response curves of etoxazole and other insecticides against *Spodoptera exigua* (resistant). 99% etoxazole (A), 5% etoxazole EC *(O),* 97% hexaflumuron ( **■** ), 98% chlorfluazuron ( **▲** ), and 5% chlorfluazuron EC (♦). High quality figures are available online.

The concentration-mortality curves as the bioassay results of these pesticides are presented in Figures 2 and 3 . The LC _50_ values and the slope ± SEM of these pesticides were calculated according to the bioassay concentration-response curve and are shown in Tables 1 and 2 . Table 1 shows that etoxazole has potent insecticidal activity against the susceptible *S. exigua* , having a greater toxicity than hexaflumuron and chlorfluazuron. Furthermore, the toxicity of etoxazole EC was greater than that of etoxazole. The commercial 5% chlorfluazuron EC was tested for its insecticidal activity and compared with 5% etoxazole EC. It was found that the toxicity of 5% etoxazole EC against *S. exigua* greater than that of chlorfluazuron EC.

**Table 1.**
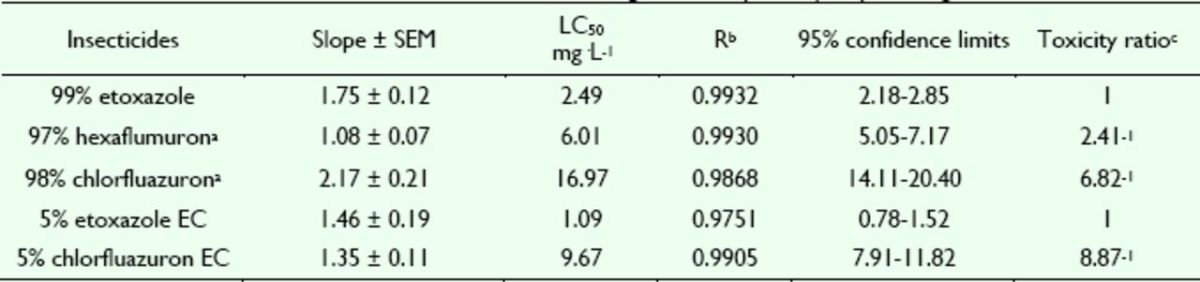
Insecticidal activities of etoxazole and other insecticides against susceptible *Spodoptera exigua.*

^a^ Hexaflumuron and chlorfluazuron were the main benzoylphenylurea chitin biosynthesis inhibitors widely used in vegetables in China, used here as contrast pesticides.

^b^ R, correlative coefficient.

^c^ Toxicity ratio = ratio of LC50 values, e.g. 2.41 = the value of 97% hexaflumuron / that of 99% etoxazole.

**Table 2.**
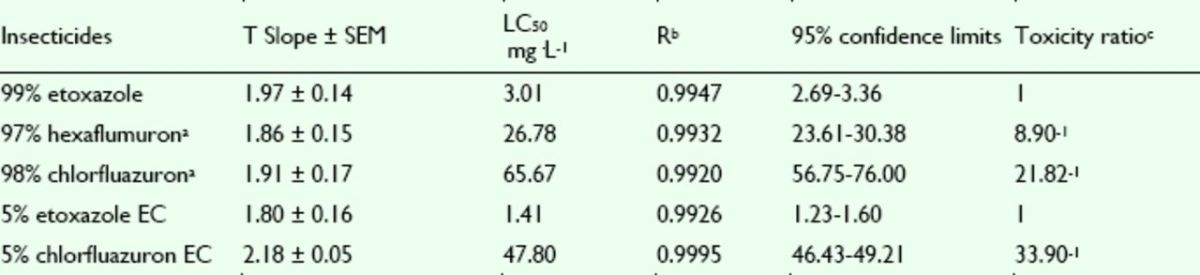
Insecticidal activities of etoxazole and other insecticide s against resistant *Spodoptera exigua.*

^a^ Hexaflumuron and chlorfluazuron were the main benzoylphenylurea chitin biosynthesis inhibitors widely used in vegetables in China, used here as contrast pesticides.

^b^ R correlative coefficient.

^c^ Toxicity ratio = ratio of LC50 values, e.g. 8.90 = the value of 97% hexaflumuron / that of 99% etoxazole.

The LC _50_ values of etoxazole, hexaflumuron, chlorfluazuron, 5% etoxazole EC, and 5% chlorfluazuronEC against the resistant strain of *S. exigua* are shown in Table 2 , which shows that the resistant strain had almost no resistance against etoxazole and 5% etoxazole EC; however, it was resistant to the other pesticides. The toxicity of etoxazole against the resistant strain was greater than that of those of hexaflumuron and chlorfluazuron. Accordingly, the toxicity of 5% etoxazole EC greater than that of 5% chlorfluazuron EC.

### Effects of insecticides against *P. xylostella*

**Figure 4.**
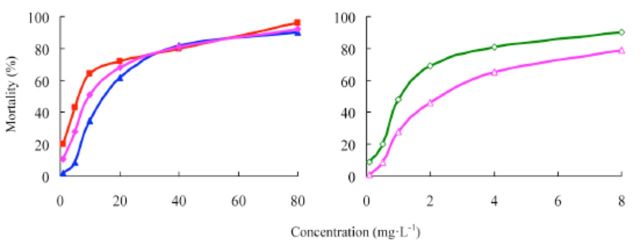
Concentration-response curves of etoxazole and other insecticides against *Plutella xylostella* (susceptible). 99% etoxazole (A), 5% etoxazole EC *(O),* 97% hexaflumuron ( **■** ), 98% chlorfluazuron ( **▲** ), and 5% chlorfluazuron EC (♦). High quality figures are available online.

**Figure 5.**
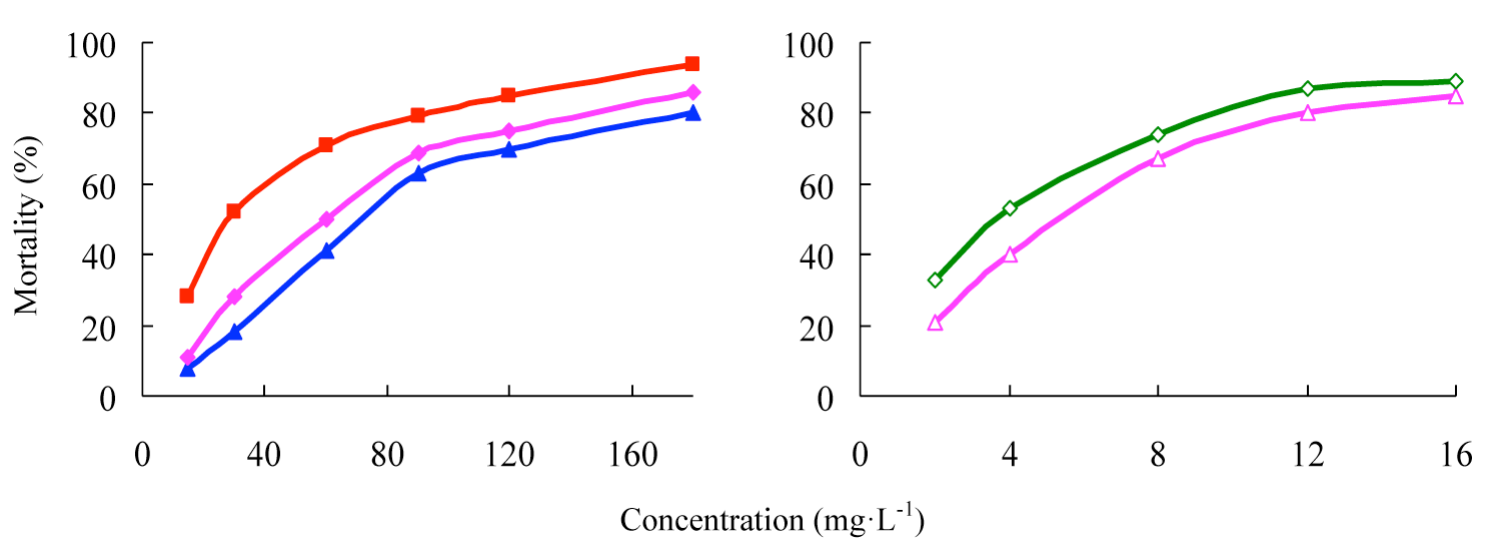
Concentration-response curves of etoxazole and other insecticides against *Plutella xylostella* (resistant). 99% etoxazole (A), 5% etoxazole EC (O), 97% hexaflumuron ( **■** ), 98% chlorfluazuron ( **▲** ), and 5% chlorfluazuron EC (♦). High quality figures are available online.

The concentration-mortality curves as the bioassay results of these pesticides are presented in Figures 4 and 5 . The LC _50_ values and the slope ± SEM of these pesticides were calculated according to the bioassay concentration-response curve and are presented in Tables 3 and 4 . The LC _50_ value of etoxazole was less than the LC _50_ values of hexaflumuron and chlorfluazuron against *P. xylostella* ( Table 3 ). Hence, the toxicity of etoxazole was greater than the toxicities of hexaflumuron and chlorfluazuron. The toxicity of 5% etoxazole was greater than that of etoxazole. The commercial 5% chlorfluazuron EC was tested for its insecticidal activity compared with 5% etoxazole EC. It was found that the toxicity of 5% chlorfluazuron EC against *P. xylostella* was greater than that of 5% chlorfluazuron EC.

**Table 3.**
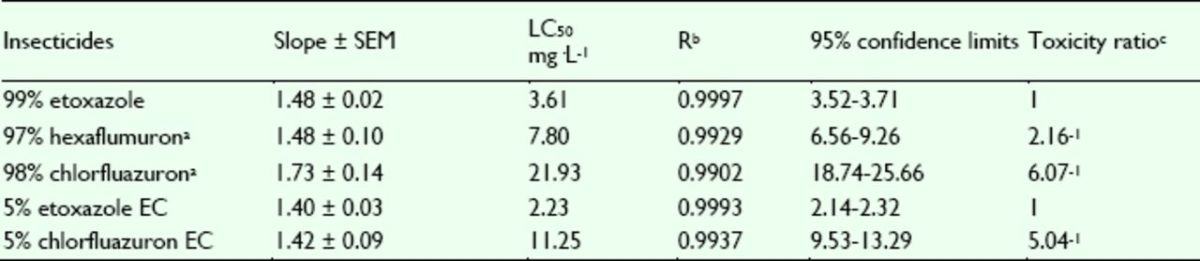
Insecticidal activities of etoxazole and other insecticide s against susceptible *Plutella xylostella.*

^a^ Hexaflumuron and chlorfluazuron were the main benzoylphenylurea chitin biosynthesis inhibitors widely used in vegetables in China, used here as contrast pesticides.

^b^ R, correlative coefficient.

^c^ Toxicity ratio = ratio of LC50 values, e.g. 2.16 = the value of 97% hexaflumuron / that of 99% etoxazole.

**Table 4.**
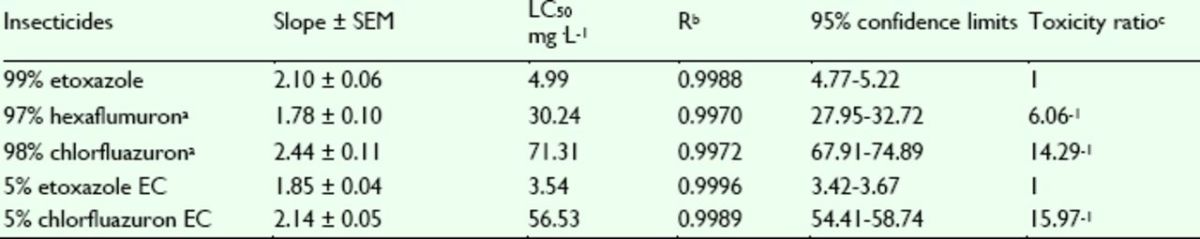
Insecticidal activities of etoxazole and other insecticides against resistant Plutella xylostella.

^a^ Hexaflumuron and chlorfluazuron were the main benzoylphenylurea chitin biosynthesis inhibitors widely used in vegetables in China, used here as contrast pesticides.

^b^ R, correlative coefficient.

^c^ Toxicity ratio = ratio of LC50 values, e.g. 6.06 = the value of 97% hexaflumuron / that of 99% etoxazole.

The LC _50_ values of etoxazole, hexaflumuron, chlorfluazuron, 5% etoxazole EC, and 5% chlorfluazuron EC against the resistant strain of *P. xylostella* are shown in Table 4 . The results show that the resistant strain had low levels of resistance to etoxazole and 5% etoxazole EC, however, they were resistant to the other pesticides. The toxicity of etoxazole against the resistant strain was greater than that of hexaflumuron and chlorfluazuron. Accordingly, the toxicity of 5% etoxazole EC was greater than that of 5% chlorfluazuron EC.

### Effects of insecticides against *A. craccivora*

**Figure 6.**
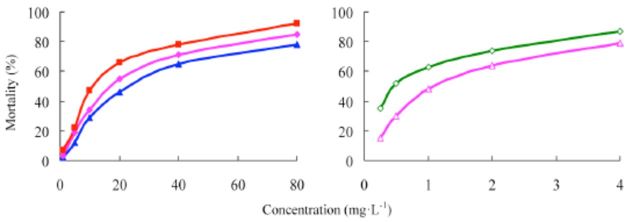
Concentration-response curves of etoxazole and other insecticides against *Aphis craccivora* (susceptible). 99% etoxazole (A), 5% etoxazole EC ( ), 95% permethrin ( **■** ), 95% acephate ( **▲** ), and 30% acephate EC (♦). High quality figures areavailable online.

**Figure 7.**
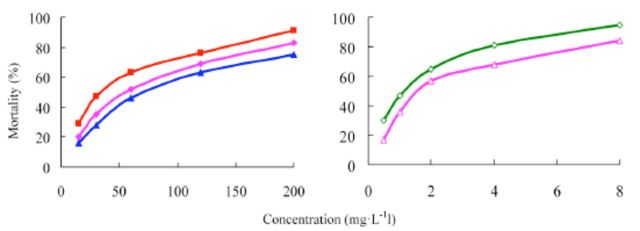
Concentration-response curves of etoxazole and othe r insecticides against *Aphis craccivora* (resistant). 99% etoxazole (A), 5% etoxazole EC (O), 95% permethrin ( **■** ), 95% acephate ( **▲** ), and 30% acephate EC (♦). High quality figures are available online.

The mortality rates of etoxazole, permethrin, and acephate against the susceptible strain of *A. craccivora* were assayed. The concentration-mortality curves as the bioassay results of these pesticides are presented in Figures 6 and 7 . The LC _50_ values and the slope ± SEM of these pesticides were calculated according to the bioassay concentration-response curve and are presented in Tables 5 and 6 . The toxicity of etoxazole was greater than the toxicities of permethrin and acephate. Hence, etoxazole has high insecticidal activity against *A. craccivora* . The toxicity of 5% etoxazole EC against *A. craccivora* was greater than that of etoxazole. The commercial 30% acephate EC was bioassayed for its insecticidal activity compared to 5% etoxazole EC. It was found that the toxicity 30% acephate EC was less than that of of 5% etoxazole EC.

**Table 5.**
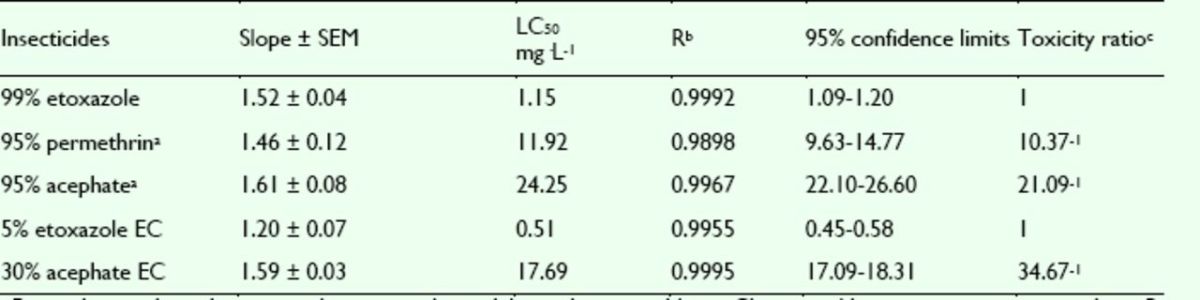
Insecticidal activities of etoxazole and other insecticides against susceptible *Aphis craccivora.*

^a^ Permethrin and acephate were the insecticides widely used in vegetables in China, used here as contrast insecticides.

^b^ R correlative coefficient.

^c^ Toxicity ratio = ratio of LC50 values, e.g. 10.37 = the value of 95% permethrin / that of 99% etoxazole.

**Table 6.**
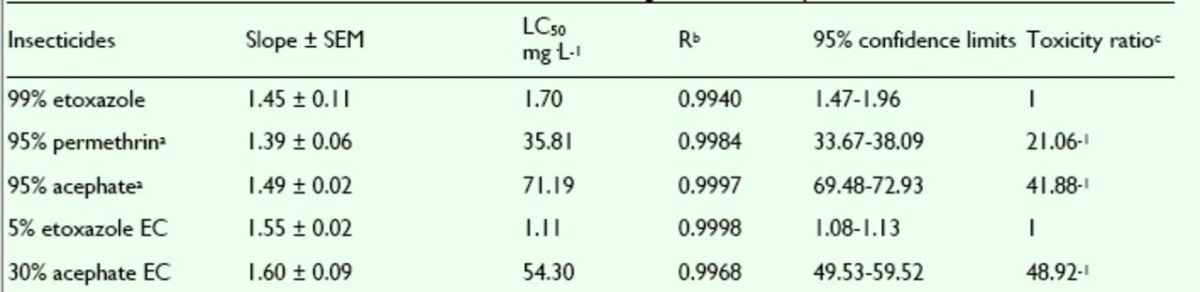
Insecticidal activities of etoxazole and other insecticides against resistant *Aphis craccivora.*

^a^ Permethrin and acephate were the insecticides widely used in vegetables in China, used here as contrast insecticides.

^b^ R correlative coefficient.

^c^ Toxicity ratio = ratio of LC50 values, e.g. 21.06 = the value of 95% permethrin / that of 99% etoxazole.

The LC _50_ values of etoxazole, permethrin, acephate, 5% etoxazole EC, and 30% acephate EC against the resistant strain of *A. craccivora* are shown in Table 6 . It is evident from these data that the resistant aphid strain was not resistant to etoxazole and 5% etoxazole EC; however, it was resistant to the other pesticides. The toxicity of etoxazole against *A. craccivora* was greater than the toxicities of permethrin and acephate. Accordingly, the toxicity of 5% etoxazole EC was great than that of 30% acephate EC.

### Effects of pesticides against *T. cinnabarinus* nymphs

**Figure 8.**
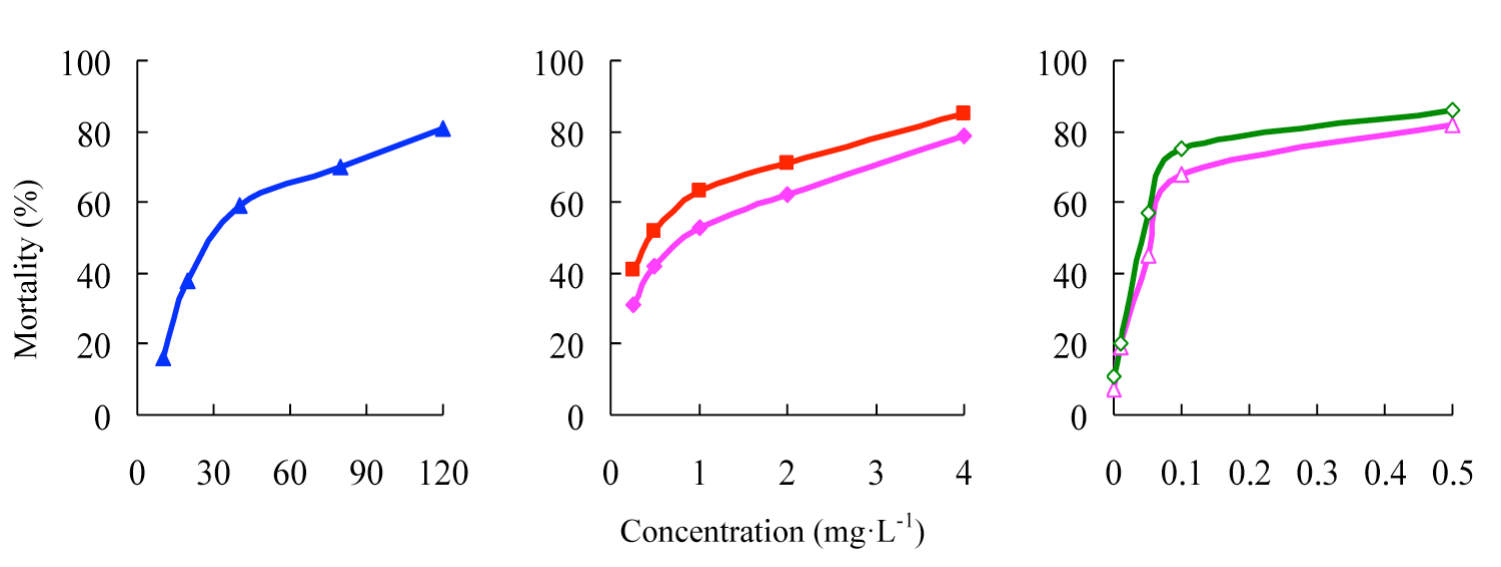
Concentration-response curves of etoxazole and othe r insecticides against *Tetranychus cinnabarinus* nymphs (susceptible). 99% etoxazole (A), 5% etoxazole EC (O), 5% hexythiazox EC ( **■** ), 98% clofentezine ( **▲** ), and 98% hexythiazox (♦). High quality figures are available online.

**Figure 9.**
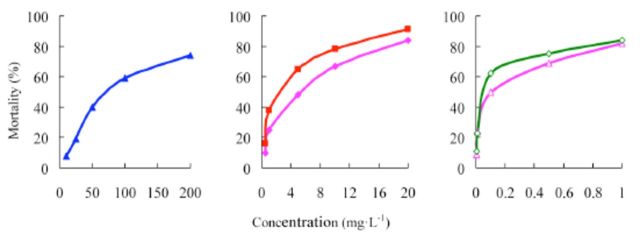
Concentration-response curves of etoxazole and other insecticides against *Tetranychus cinnabarinus* nymphs (resistant). 99% etoxazole (A), 5% etoxazole EC (O), 5% hexythiazox EC ( **■** ), 98% clofentezine ( **▲** ), and 98% hexythiazox (♦). High quality figures are available online.

The mortality rates of etoxazole, hexythiazox, and clofentezine against nymphs of the *T. cinnabarinus* were assayed. The concentration-mortality curves as the bioassay results of these pesticides are shown in Figures 8 and 9 . The LC _50_ values and the slope ± SEM of these pesticides were calculated according to the bioassay concentration-response curve and are given in Tables 7 and 8 . The LC _50_ value of etoxazole was less than the LC _50_ values of hexythiazox and clofentezine against *T. cinnabarinus* nymphs ( Table 7 ). The toxicity of etoxazole was greater than the toxicities of hexythiazox and clofentezine. The toxicity of 5% etoxazole EC was greater than that of etoxazole. The commercial 5% hexythiazox EC was tested for its acaricidal activity and compared with 5% etoxazole EC. It was found that the toxicity of 5% hexythiazox EC was less than that of 5% etoxazole EC.

**Table 7.**
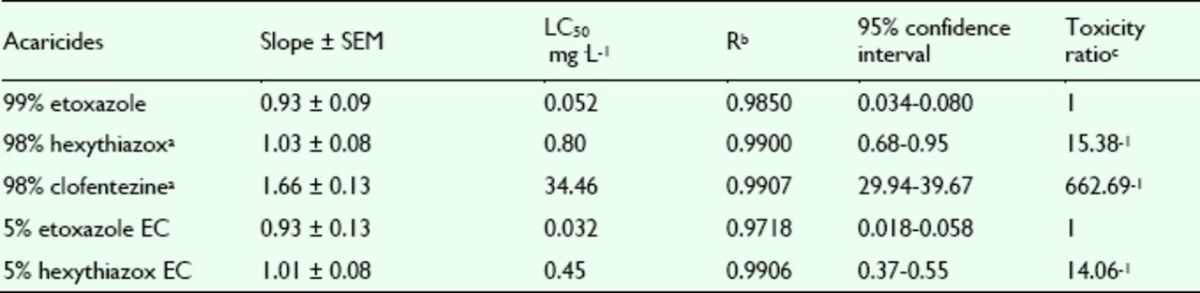
Acaricidal activities of etoxazole and other acaricides ag ainst susceptible *Tetranychus cinnabarinus* nymphs.

^a^ Hexythiazox and clofentezine were the acaricides widely used in vegetables in China, used here as contrast acaricides.

^b^ R, correlative coefficient.

^c^ Toxicity ratio = ratio of LC50 values, e.g. 1 5.38 = the value of 98% hexythiazox / that of 99% etoxazole.

**Table 8.**
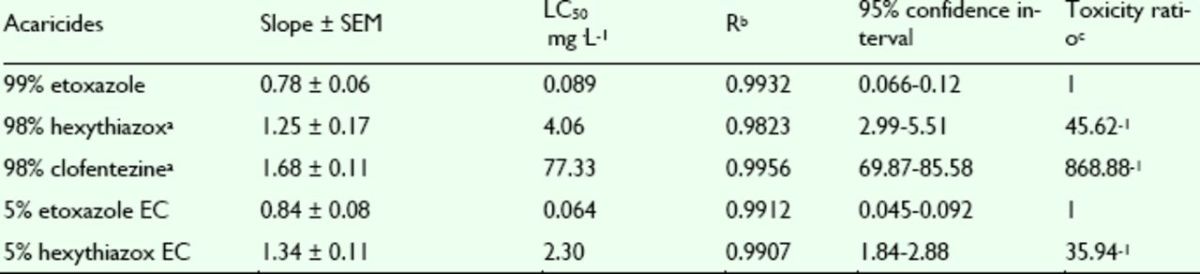
Acaricidal activities of etoxazole and other acaricides ag ainst resistant *Tetranychus cinnabarinus* nymphs.

^a^ Hexythiazox and clofentezine were the acaricides widely used in vegetables in China, used here as contrast acaricides.

^b^ R, correlative coefficient.

^c^ Toxicity ratio = ratio of LC50 values, e.g. 45.62 = the value of 98% hexythiazox / that of 99% etoxazole.

The LC _50_ values of etoxazole, hexythiazox, clofentezine, 5% etoxazole EC, and 5% hexythiazox EC against the resistant strain of *T. cinnabarinus* are shown in Table 8 . It is evident that the resistant strain had almost no resistance against etoxazole and 5% etoxazole EC; however, it was resistant against the other pesticides. The toxicity of etoxazole against resistant *T. cinnabarinus* was greater than the toxicities of hexythiazox and clofentezine. Accordingly, the toxicity of 5% etoxazole EC was great than that of 5% hexythiazox EC.

### Effects of pesticides against *T. cinnabarinus* eggs

**Figure 10.**
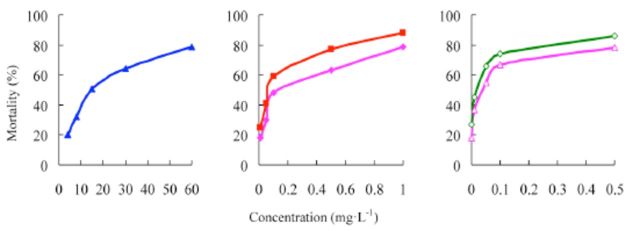
Concentration-response curves of etoxazole and other insecticides against *Tetranychus cinnabarinus* eggs (susceptible). 99% etoxazole (△), 5% etoxazole EC ( **◇** ), 5% hexythiazox EC ( **■** ), 98% clofentezine ( **▲** ), and 98% hexythiazox ( **◆** ). High quality figures are available online.

**Figure 11.**
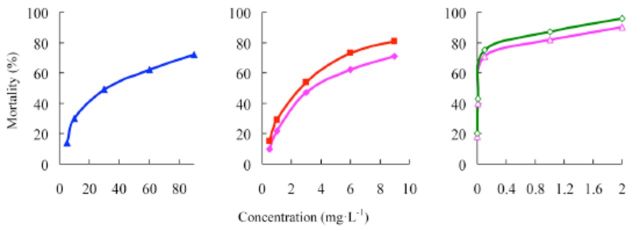
Concentration-response curves of etoxazole and other insecticides against *Tetranychus cinnabarinus* eggs (resistant). 99% etoxazole (△), 5% etoxazole EC ( **◇** ), 5% hexythiazox EC ( **■** ), 98% clofentezine ( **▲** ), and 98% hexythiazox ( **◆** ). High quality figures are available online.

The effects of etoxazole, hexythiazox, and clofentezine against eggs of the susceptible strain of the mite *T. cinnabarinus* were assayed. The concentration-mortality curves as the bioassay results of these acaricides are shown in Figures 10 and 11 . The LC _50_ values and the slope ± SEM of these pesticides were calculated according to the bioassay concentration-response curve and are presented in Tables 9 and 10 . The LC _50_ value of etoxazole was les than the LC _50_ values of hexythiazox and clofentezine against spider mite eggs ( Table 9 ). The toxicity of etoxazole was greater than the toxicities of hexythiazox and clofentezine. The toxicity of 5% etoxazole EC was greater than that of etoxazole. The commercial 5% hexythiazox EC was tested for its acaricidal activity and compared with 5% etoxazole EC. It was found that the toxicity of 5% etoxazole EC against spider mite eggs was great than that of 5% hexythiazox EC.

**Table 9.**
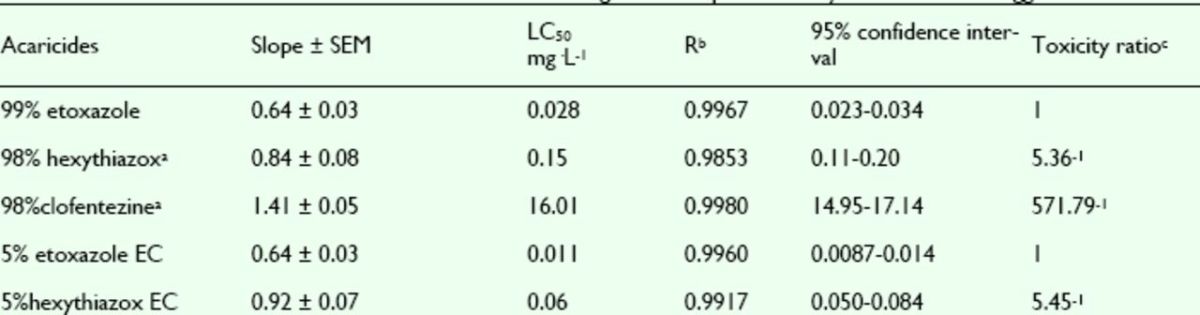
Acaricidal activities of etoxazole and other acaricides against susceptible *Tetranychus cinnabarinus* eggs.

^a^ Hexythiazox and clofentezine were the acaricides widely used in vegetables in China, used here as contrast acaricides.

^b^ R, correlative coefficient.

^c^ Toxicity ratio = ratio of LC50 values, e.g. 5.36 = the value of 98% hexythiazox / that of 99% etoxazole.

**Table 10.**
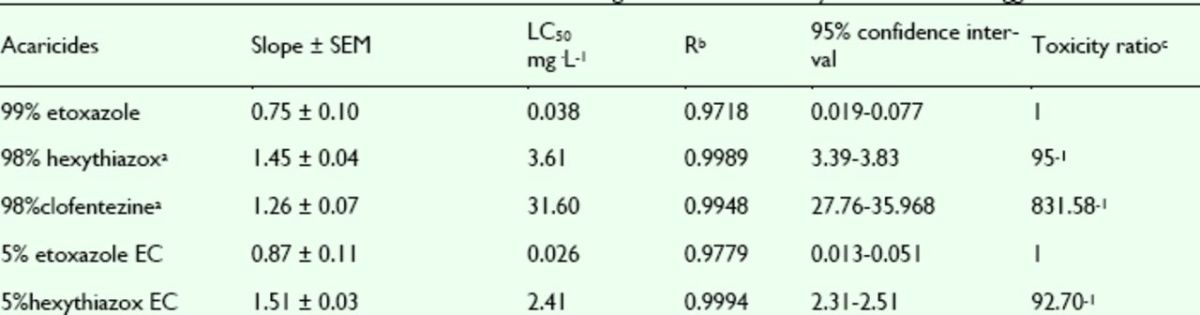
Acaricidal activities of etoxazole and other acaricides against resistant *Tetranychus cinnabarinus* eggs.

a Hexythiazox and clofentezine were the acaricides widely used in vegetables in China, used here as contrast acaricides. b R, correlative coefficient. c Toxicity ratio = ratio of LC50 values, e.g. 95 = the value of 98% hexythiazox / that of 99% etoxazole.

The LC _50_ effects of etoxazole, hexythiazox, clofentezine, 5% etoxazole EC, and 5% hexythiazox EC against the resistant strain of *T. cinnabarinus* are shown in Table 10 . It is evident that the resistant strain had almost no resistance to etoxazole and 5% etoxazole EC; however, it was resistant against the other pesticides. The toxicity of etoxazole against spider mite nymphs from the field was greater than the toxicities of hexythiazox and clofentezine. Accordingly, the toxicity of 5% etoxazole EC was greater than that of 5% hexythiazox EC.

In conclusion, it was seen that etoxazole is an effective insecticide/acaricide. On the basis of bioactive results, 5% etoxazole EC was an excellent formulation alternative to etoxazole applied in vegetable fields. Furthermore, etoxazole combined with other insecticides/acaricides in vegetables can be used to achieve integrated pest management. Consequently, etoxazole is a suitable biorational alternative to traditional, highly toxic pesticides, with important significance to vegetable crop protection from insects and acariin China.

## Abbreviations

DMSO: dimethyl sulfoxide
EC: emulsifiable concentrate
